# Meta-analysis of prediction model performance across multiple studies: Which scale helps ensure between-study normality for the *C*-statistic and calibration measures?

**DOI:** 10.1177/0962280217705678

**Published:** 2017-05-08

**Authors:** Kym IE Snell, Joie Ensor, Thomas PA Debray, Karel GM Moons, Richard D Riley

**Affiliations:** 1 Research Institute for Primary Care and Health Sciences, Keele University, Staffordshire, UK; 2 Julius Center for Health Sciences and Primary Care, University Medical Center Utrecht, Utrecht, The Netherlands; 3 Cochrane Netherlands, University Medical Center Utrecht, Utrecht, The Netherlands

**Keywords:** Validation, performance statistics, *C-*statistic, discrimination, calibration, meta-analysis, between-study distribution, heterogeneity, simulation

## Abstract

If individual participant data are available from multiple studies or clusters, then a prediction model can be externally validated multiple times. This allows the model’s discrimination and calibration performance to be examined across different settings. Random-effects meta-analysis can then be used to quantify overall (average) performance and heterogeneity in performance. This typically assumes a normal distribution of ‘true’ performance across studies. We conducted a simulation study to examine this normality assumption for various performance measures relating to a logistic regression prediction model. We simulated data across multiple studies with varying degrees of variability in baseline risk or predictor effects and then evaluated the shape of the between-study distribution in the *C-*statistic, calibration slope, calibration-in-the-large, and E/O statistic, and possible transformations thereof. We found that a normal between-study distribution was usually reasonable for the calibration slope and calibration-in-the-large; however, the distributions of the *C-*statistic and E/O were often skewed across studies, particularly in settings with large variability in the predictor effects. Normality was vastly improved when using the logit transformation for the *C*-statistic and the log transformation for E/O, and therefore we recommend these scales to be used for meta-analysis. An illustrated example is given using a random-effects meta-analysis of the performance of QRISK2 across 25 general practices.

## 1 Introduction

Clinical prediction models aim to guide clinical decision-making by accurately predicting the probability of an outcome in patients, for either diagnostic (i.e. to predict existing outcomes, usually disease presence) or prognostic purposes (i.e. to predict future outcomes).^1,2^ Prediction models are often developed using multivariable regression analysis, and typically include a combination of two or more variables (‘predictors’) to predict the outcome. Statistically, for a prediction model to perform well, it should have good calibration and discrimination.^3,4^ Discrimination of the model is its ability to distinguish between patients who have the outcome of interest and those that do not. Calibration is how well the predicted probabilities from the model agree with the observed outcome frequencies in the data.

Prediction models often perform well in the data used to develop the model, but worse when applied to new subjects.^1,3,5^ Therefore, it is strongly recommended to externally validate prediction models using independent data, not used for the development.^6–8^ Ideally, a model should be externally validated in various samples, to evaluate its potential generalisability and need for re-calibration or other model updating.^8–10^ This is possible when multiple sources of data are available, such as individual participant data (IPD) from different countries, studies or clusters (e.g. centres in registry data).^11–13^

Recently, random-effects meta-analysis has been recommended to summarise predictive performance measures of a model that have been validated across multiple studies or clusters,^14–17^ and to assess the presence of unexplained (statistical) heterogeneity in these measures. It has previously been shown that between-study heterogeneity is highly likely due to changes in case mix variation, baseline risk, and predictor effects.^15,17–20^ For this reason, evaluating the impact of such heterogeneity is crucial to fully appraise the summary performance of a developed prediction model and to evaluate its potential generalisability. In this regard, prediction intervals offer a valuable asset, as these intervals describe the likely range of model performance in a new population or setting similar to one of those included in the analysed studies. Hereto, they account for imprecision of the summary performance estimates and for heterogeneity (variability) of ‘true’ performance across studies.

A standard model for random-effects meta-analysis assumes that the ‘true’ performance is normally distributed within and across studies.^21,22^ When studies are reasonably large, within-study normality of performance estimates can be justified by appealing to the central limit theorem. In particular, maximum likelihood estimates are known to be approximately normally distributed. However, in terms of the between-study distribution of true performance, the assumption of normality is usually based on convenience, rather than any sound reasoning.^23^ Yet, the assumption is fundamentally important when making predictive inferences about potential performance in new studies or populations; for example, when deriving a 95% prediction interval for the potential calibration slope in a new study.^14,22^

Lee and Thomson explored flexible parametric models for random-effects meta-analysis when the normality assumption between studies does not hold, and highlight that it is important to allow for skewing, especially when the predictive distribution is of interest.^24^ In a novel case study, Van Klaveren et al. showed the importance of random effects meta-analysis for examining the discrimination performance of a prediction model in clustered data, and considered the distributional assumption when pooling *C-*statistics across clusters.^14^ Plotting standardised residuals for both the original C-statistic scale and the logit scale, the authors concluded that the original *C*-statistic scale was the most appropriate in their particular example.However, in general they recommend that to ‘decide if the meta-analysis should be undertaken on the probability scale or the log-odds scale we suggest considering the normality assumptions on both scales by normal probability plots and Shapiro-Wilk tests of the standardized residuals’. Though this can be helpful, it may be difficult to disentangle the within-study variability (sampling error of study-specific *C-*statistic estimates) from the between-study variability (heterogeneity in the true *C*-statistic across studies, e.g. due to differences in case-mix). Furthermore, when the number of studies is small, it is hard to ascertain whether observed discrepancies with normality are genuine or simply due to chance. In such situations, it is also helpful to consider external empirical evidence, for example about the between-study distribution of the *C*-statistic and logit *C*-statistics observed in large-scale simulation studies. Simulation also allows multiple performance statistics to be evaluated, for example to summarise calibration performance, for which normality of between-study distributions is also of interest.

This motivates us, therefore, to perform an extensive simulation study to examine the true between-study distribution of the *C-*statistic and other model performance measures in a wide set of situations. We do this through simulated individual participant data from multiple studies across a variety of realistic settings, with large study sample sizes used to minimise within-study sampling error. Our objective is to identify appropriate scales to use for meta-analysing the *C-*statistic and also calibration measures (calibration slope, calibration-in-the-large, and the ratio of expected to observed (E/O) probabilities), such that the true between-study distribution of the performance statistic is likely to be approximately normal.

The outline of the paper is as follows. Section 2 provides background details on model performance measures and random-effects meta-analysis. Section 3 describes the simulation study design, scenarios and results. Section 4 then illustrates the key finding using a random-effects meta-analysis to summarise the performance of the QRISK2 model across multiple general practices.^25^ Section 5 concludes with discussion, including recommendations for the scale on which to pool the *C-*statistic and calibration measures.

## 2 Meta-analysis to summarise predictive performance of a prediction model

Let us consider a simple prediction model developed for a binary outcome (either diagnostic, or prognostic occurring in a relatively short time period) using a logistic regression model with a single predictor. The fitted model can be written as

(1) logit(p^i)=α^+β^xi

where *x_i_* is the predictor value for patient *i*. The predicted probability of the outcome for a patient can be calculated as

(2) p^i=eLPi1+eLPi

where *LP_i_* =  α^+β^xi and is referred to as the linear predictor.^26^ As mentioned, it is important to externally validate the performance of such a model across multiple data samples or sources. We now introduce definitions of key performance statistics, and then outline the standard model for random-effects meta-analysis to summarise performance statistics of a specific model estimated from multiple validation studies.

### 2.1 Measures of model performance considered

When validating a prediction model, it is important to consider both the discrimination and calibration performance of the model.

#### 2.1.1 Discrimination

Discrimination is a measure of how well a model can differentiate between individuals who have the outcome of interest and those that do not. The *C-*statistic is a commonly used measure of discrimination and is calculated as the proportion of all possible pairs (of individuals in which one had the outcome of interest and the other did not) that are concordant. A pair is said to be concordant if the predicted probability is higher for the individual that had the outcome compared to the predicted probability for the individual that did not.^26^ This is equivalent to the area under the ROC curve for a logistic regression model.^27^ The closer the *C*-statistic is to the value 1, the better the model discriminates between individuals that did and did not have the outcome. A value of 0.5 suggests that the model predicts no better than chance alone.

#### 2.1.2 Calibration

The calibration of a model is a measure of how well the predicted probabilities from the model agree with the observed outcome frequencies or probabilities. Calibration statistics typically quantify how much the model under- or over-predicts outcome risk, ideally across the spectrum of predicted risks (often defined by tenths of predicted risks).

The calibration statistics considered in this paper are:

- **Calibration-in-the-large:** This is the difference between the mean number of predicted outcomes and the mean number of observed outcomes.^1,28^ A calibration model written as
  (3) $$\mathrm{logit}(p_{i})=\alpha +\beta ({\mathrm{LP}}_{i})$$
  can be fitted where the estimate of *α* given *β* = 1 (*LP_i_* is used as an offset) provides the estimate of calibration-in-the-large.^1^ Calibration-in-the-large should be close to zero for a well-calibrated model.
- **Expected/observed ratio (E/O):** Another common summary measure of overall calibration is given by the ratio of expected (E) and observed (O) total number of outcomes (or ratio of the E and O outcome probabilities). This quantity should be close to one if the model calibrates well in the validation dataset, and is directly related to the calibration-in-the-large statistic.^17^
- **Calibration slope:** If the calibration model given in equation (3) is fitted without constraints, β^ is the estimated calibration slope.^20,29^ A calibration slope <1 indicates overfitting, where predictions from the model are more extreme than the observed outcomes in the validation dataset (predictions for high risk patients are too high and predictions for low risk patients are too low). A calibration slope >1 indicates underfitting, where predictions do not vary enough (predictions for high risk patients are not high enough and predictions for low risk patients are not low enough). Overfitting is more common (e.g. due to automated selection procedures); however, underfitting can occur, e.g. when predictor effects were overly shrunk using penalisation methods during the model development. A model that calibrates well in the validation dataset would result in a calibration slope close to 1.

### 2.2 Random-effects meta-analysis for summarising performance across studies

Consider now that we wish to meta-analyse a particular performance statistic (e.g. *C*-statistic) across multiple validation studies. The standard, random-effects meta-analysis can be written as

(4) Y i∼N(μi,Si2),μi∼N(μ,τ2)

where *Y_i_* is the performance statistic estimated in study *i* = 1, … , *k*. The meta-analysis assumes normality of the performance statistic, both at the within-study and between-study levels. Within each study, the estimated performance statistic is assumed to be normally distributed about some true performance for that study (*μ_i_*) with ‘known’ variance ( $S_{i}^{2}$ ). Between studies, the true performance statistic from each study is also assumed to be drawn from a normal distribution with mean performance *μ* and between-study variance τ ^2^.

#### 2.2.1 Confidence intervals

Following estimation of a meta-analysis model, an approximate 100(1−*α*)% confidence interval for the mean pooled performance statistic *μ* can be estimated using the Hartung–Knapp–Sidik–Jonkman (HKSJ) approach which is recommended over conventional calculations, particularly when the number of studies, *k*, is small and when between-study heterogeneity is likely to be present.^30–32^ This can be calculated as

(5) μ^±tk-1;α2qσ^μ

using the 1−α/2 quantile of the Student’s *t*-distribution with *k*−1 degrees of freedom, multiplied by $\sqrt{q}$ and the estimated standard error of μ^ , σ^μ

q=1k-1∑iw^i(yi-μ^)2;σ^μ=1∑iw^i

where the estimated weights are calculated as

w^i=1S^i2+τ^2.

#### 2.2.2 Prediction intervals

A random-effects meta-analysis (model (4)) assumes a normal distribution of true performance across studies. Therefore, the performance in a single study may differ considerably from the average performance. To address this, Higgins et al. propose that an approximate 100(1−*α*)% prediction interval for model performance in a new but similar study can also be derived following random-effects meta-analysis (4), using the formula

(6) μ^±tα,k-2τ^2+σ^μ2

where $t_{\alpha ,k-2}$ is the $100(1-\frac{\alpha }{2})\%$ percentile of the t-distribution for *k*−2 degrees of freedom.^22^ The *t*-distribution is used to account for τ^2 being an estimate itself and therefore having uncertainty that is otherwise not accounted for. This frequentist-based formulae is only an approximation, but gives an indication as to the range of performance a model may have over relevant populations and settings.^33^ A Bayesian approach to derive predictive intervals and distributions is also possible and is a more natural framework.^22^ Regardless, the assumption of between-study normality is always important when deriving predictive inferences based on model (4).

#### 2.2.3 Normality assumption within and between studies

The assumption of within-study and between-study normality is common in the meta-analysis field.^21,22^ As noted earlier, the assumption of within-study normality (of the *Y_i_*’s) is reasonable if the sample size and number of subjects with the outcome in the validation study are reasonably large, as one can then appeal to the Central Limit Theorem (CLT). The assumed between-study normality of the true performance statistics (*μ_i_*’s) across studies is harder to justify. Baker and Jackson are critical of this assumption in random-effects meta-analysis models in general, as it is often based on pragmatic rather than theoretical reasons. They state: ‘the CLT does not really imply anything for the distribution of the random effects… We can only appeal to the CLT here with the vague idea that the unknown source of variation between studies might be the sum of several factors’.^23^

In Section 3, we investigate (through a simulation study) the best scale to meta-analyse performance statistics so that their between-study distribution is more likely to be normally distributed. We assume that within-study normality is justified by studies being reasonably large. Further work may also examine within-study distributions, but our focus here is on the validity of the between-study normal distribution. We have explicitly designed our simulation study to disentangle within-study and between-study distributions, essentially by making within-study variability tiny.

#### 2.2.4 Alternative scales for meta-analysis of performance measures

If the between-study distribution is not normally distributed for any of the performance measures on their original scale, it may be more appropriate to transform the measurements to an alternative scale for meta-analysis. For example, if using a natural logarithm transformation, the random-effects model can be written as

(7) $$\log(Y_{i})∼N(\mu_{i},S_{i}^{2}),\mu_{i}∼N(\mu ,\tau^{2})$$

where log stands for the natural logarithm. Assuming normality at both levels of a transformed variable is easier to implement in a meta-analysis than assuming normality on different scales of the variable. In this simulation study, we are interested in the most appropriate scale for the between-study distribution, and therefore minimise within-study error to reveal the true between-study distribution.

We will consider the natural logarithm (log) transformation which often works well for ratios that are subject to right skewing (such as hazard ratios and odds ratios). The logit transformation is often used for proportions such as probabilities (e.g. in a logistic regression model) and showed good coverage for confidence intervals of the area under the ROC curve (same as the *C*-statistic) for diagnostic tests in a simulation study by Qin and Hartilovac.^34^ The logit transformation for the *C*-statistic is calculated as log(*C_i_*/(1 − *C_i_*)). We will also consider the variance stabilising transformations suggested by Trikalinos et al., which are the arcsine and square root transformations for proportions and rates, respectively.^35^

## 3 Simulation study

### 3.1 Methods for simulation

In all simulations, we consider the performance of a chosen prediction model across multiple validation studies. The assumed prediction model to be examined was considered to be a logistic regression model for a binary outcome, and initially we consider that there is just a single continuous variable (predictor) in the model. Building on previous work,^19^ the clinical example considered is a diagnostic model for diagnosis of deep vein thrombosis (DVT), with (initially) age as the only predictor. This logistic regression prediction model can be written as

(8) $$\mathrm{logit}(p_{\mathrm{ij}})=\mu_{\alpha }+\mu_{\beta }\times {\mathrm{age}}_{\mathrm{ij}}$$

where *i* represents the individual in study *j*.

For each simulation scenario, the performance of this model is examined across validation studies, covering settings where the model is correctly specified for each study (no variability) and settings where it is correct on average but not in all studies (between-study variability). We begin with base scenarios in which the model is correctly specified in all studies and thus variability in model performance only appears due to sampling error. Other settings are then defined in which the prediction model performance may vary across studies due to between-study variability in ‘true’ predictor effects.

#### 3.1.1 Defining scenarios for simulation

##### Nine base scenarios

We considered the example of predicting risk of deep vein thrombosis (DVT) using a single predictor such as age to define the first base scenario. Values from Oudega et al. informed the distribution of age, predictor effect of age and the model intercept (using the prevalence of DVT).^36^ Therefore, we generated patient data assuming mean-centred age ∼ Normal(0,17.6^2^). A univariable odds ratio of 1.01 was reported for age (which relates approximately to μ_β_ = 0.01) and the prevalence of DVT was taken as 0.22, which can be achieved by generating data with μ_α_ = −1.274, given that μ_β_ = 0.01.

Eight other base scenarios were also defined by varying the parameter values of μ_α_ and μ_β_ to give a different prevalence and/or strength of the predictor. Parameter values were selected as if all generated studies come from exactly the same population (generated using the same true prediction model). The nine scenarios are defined in Table 1.

**Table 1.** Parameter values of the true prediction model in nine base scenarios.

Scenario	μ_α_	μ_β_	Prevalence^a^	*C*-statistic^a^
1	−1.274	0.010	0.22	0.55
2	−2.957	0.010	0.05	0.55
3	2.210	0.010	0.90	0.55
4	−1.425	0.045	0.22	0.7
5	−3.215	0.045	0.05	0.7
6	2.440	0.045	0.90	0.7
7	−2.386	0.145	0.22	0.9
8	−5.133	0.145	0.05	0.9
9	3.987	0.145	0.90	0.9

a The μ_α_ and μ_β_ values are selected to give the corresponding average prevalence and *C*-statistic (average from 100 large samples each of 1,000,000 patients).

##### Size and number of studies selected to assess between-study distributions

In order to examine the shape of the between-study distributions in model performance, within-study sampling error should be minimised. For this reason, we generated very large study samples so as to avoid the amalgamation of the within-study and between-study distributions. Based on preliminary simulations, we decided to use 1000 studies each containing 500,000 patients (Supplemental material 1).

##### Simulation settings introducing variability between studies

The nine base scenarios were extended to a variety of simulation settings in which variability was introduced for either the intercept term or the predictor effect when generating the patient-level data. Seven such simulation settings were defined (Table 2). In each of these settings, the models used to generate the study level data were defined using a study-specific intercept and a study-specific predictor effect, *α_j_* and *β_j_*, respectively. The models used to generate data can now be written as

(9) $$\begin{array}{c} \mathrm{logit}(p_{\mathrm{ij}})=\alpha_{j}+\beta_{j}\times {\mathrm{age}}_{\mathrm{ij}}\mathrm{where}\alpha_{j}∼\mathrm{Normal}(\mu_{\alpha },\sigma_{\alpha }^{2});\beta_{j}∼\mathrm{Normal}(\mu_{\beta },\sigma_{\beta }^{2}). \end{array}$$

**Table 2.** Defined settings for simulation, with variability in either *α_j_* or *β_j_* across studies.

Simulation setting	Standard deviation for *α_j_* and *β_j_* in each scenario	
1: No variability in *α_j_* or *β_j_*	σ_α_ = 0	σ_β_ = 0
2: Little variability in *α_j_*	σ_α_ = 0.1	σ_β_ = 0
3: Moderate variability in *α_j_*	σ_α_ = 0.5	σ_β_ = 0
4: Large variability in *α_j_*	σ_α_ = 1.0	σ_β_ = 0
5: Little variability in *β_j_*	σ_α_ = 0	σ_β_ = 0.005
6: Moderate variability in *β_j_*	σ_α_ = 0	σ_β_ = 0.020
7: Large variability in *β_j_*	σ_α_ = 0	σ_β_ = 0.070

There was variability across studies in *α_j_* if $\sigma_{\alpha }>0$ and variability in *β_j_* if $\sigma_{\beta }>0$ . The prediction model being validated in all studies remained the same as before (model (8)), where the intercept is the mean of the study-specific intercepts μ_α_, and the predictor effect is the mean of the study specific predictor effects $\mu_{\beta }.$ In such situations where there is variability across studies ( $\sigma_{\alpha }>0$ or $\sigma_{\beta }>0$ ), the prediction model in (8) is incorrect, as it ignores the variability in *α_j_* or *β_j_* and therefore will induce heterogeneity in its performance when applied to different populations. Setting 1 is defined as having no variability in *α_j_* or *β_j_*, so all the study-specific intercepts are identical and equal to the prediction model intercept (μ_α_), as are the study-specific predictor effects (μ_β_). In such situations, the prediction model is correct in all populations. This setting is useful as a comparison for the other simulation settings.

For simplicity, we consider variability in either *α_j_* or *β_j_* but not for both at the same time. Three values were selected for σ_α_ and three values for σ_β_. The values were selected to give small, moderate and large variation in either *α_j_* or *β_j_* but are fairly arbitrary as the amount of heterogeneity in the performance statistic also depends on the *α_j_* and *β_j_* values themselves. The standard deviations (SDs) for *β_j_* (σ_β_'s) were chosen to be values of about half the μ_β_ used in defining the scenarios. Therefore, the largest value of σ_β_ = 0.07 is extreme for scenarios 1–3 where μ_β_ = 0.01 and large for scenarios 4–6 where μ_β_ = 0.045. The seven different simulation settings are defined in Table 2 in terms of σ_α_ and σ_β_.

In summary, we use nine simulation *scenarios* which define the prediction models in terms of the average intercept and average predictor effect. We also define seven simulation *settings* which relate to the level of variability in either the intercept or predictor effect. There are therefore 63 simulations considered (9 scenarios × 7 settings), which cover a range of values for μ_α_ and μ_β_, and different levels of variability in either *α_j_* and *β_j_*. We also define extensions to the simulation study for more realistic data situations which are discussed at the end of Section 3.1.1.

##### Generating patient-level data and calculating performance statistics

For each scenario in each setting, patient-level data (i.e. mean-centred age and binary DVT outcome) were generated for 500,000 patients in each of 1000 validation studies based on model (9). The individual patient data were generated for each validation study *j* by first sampling α*_j_* and β*_j_* values from the distributions $\alpha_{j}∼\mathrm{Normal}(\mu_{\alpha },\sigma_{\alpha }^{2})$ , $\beta_{j}∼\mathrm{Normal}(\mu_{\beta },\sigma_{\beta }^{2})$ . Mean-centred age was sampled for each patient from age*_ij_* ∼ $\mathrm{Normal}(0,17.6^{2})$ , the linear predictor was then calculated for each patient as ${\mathrm{LP}}_{\mathrm{ij}}=\alpha_{j}+\beta_{j}\times {\mathrm{age}}_{\mathrm{ij}}$ and from this the outcome probability was calculated, $p_{\mathrm{ij}}=\frac{\exp({\mathrm{LP}}_{\mathrm{ij}})}{1+\exp({\mathrm{LP}}_{\mathrm{ij}})}$ . Using a Bernoulli distribution with probability *p_ij_* , a binary outcome was sampled for each patient (outcome = 0 or 1).

For each validation study generated, the prediction model to be examined was applied (i.e. model (8) using μ_α_ and μ_β_ from Table 1) to each patient to obtain predicted probabilities, and then performance statistics calculated by quantifying discrimination (by the *C-*statistic) and calibration (by E/O, calibration-in-the-large, and calibration slope) across all patients.

##### Evaluating the between-study distributions for performance statistics

The distribution of the obtained performance statistics across the 1000 studies was summarised using the mean, SD, median, minimum, maximum and interval containing 95% of values. The coefficient of skewness and coefficient of kurtosis were also calculated to summarise the shape of the between-study distribution. Summary statistics were then compared to those expected from a normal distribution (i.e. an equal mean and median, skewness of 0 and kurtosis of less than ±3).

The between-study distributions were also plotted using histograms (with normal distribution lines using mean and SD from data overlaid). The symmetry and shape of the distribution could then visually be compared to a normal distribution.

In settings where the between-study distribution of a performance statistic was not approximately normal, transformations were applied, plotted and summarised to ascertain if they offered any improvement in the between-study normality assumption. Transformations considered were the natural logarithm, logit, arcsine (considered for the *C-*statistic) and square root (considered for E/O). The arcsine and square root transformations were considered based on the work of Trikalinos et al., who recommended them as variance stabilising when meta-analysing proportions and rates, respectively.^35^

All steps to the simulation study are summarised in Box 1.

**Box 1. Outline of the steps taken in the simulation study to evaluate between-study distributions of performance statistics.**

**Step 1:** Define the prediction model to be examined (i.e. select one of the scenarios 1–9 from Table 1 to define a prediction model based on model (8)).

**Step 2:** Define model (9) as the true model, and define the amount of between-study variability in either the study-specific intercept (α*_j_*) or predictor effect (β*_j_*) (i.e. select one of the seven settings from Table 2).

**Step 3:** For each validation study *j*, sample the true intercept (α*_j_*) and predictor effect (β*_j_*) from $\alpha_{j}∼\mathrm{Normal}(\mu_{\alpha },\sigma_{\alpha }^{2})$ and $\beta_{j}∼\mathrm{Normal}(\mu_{\beta },\sigma_{\beta }^{2})$ .

**Step 4:** Within each validation study *j* = 1,…, 1000, generate patients *i* = 1,…, 500,000 and sample mean-centred age from age*_ij_* ∼ $\mathrm{Normal}(0,17.6^{2})$ for each patient.

**Step 5:** Within each validation study *j, g*enerate the binary outcome variable for each patient by first calculating the linear predictor for each individual, ${\mathrm{LP}}_{\mathrm{ij}}=\alpha_{j}+\beta_{j}\times {\mathrm{age}}_{\mathrm{ij}}$ and using this to calculate the probability of the outcome occurring by $p_{\mathrm{ij}}=\frac{\exp({\mathrm{LP}}_{\mathrm{ij}})}{1+\exp({\mathrm{LP}}_{\mathrm{ij}})}$ . The binary outcome is then sampled from a Bernoulli distribution where ${\mathrm{outcome}}_{\mathrm{ij}}∼\mathrm{Bernoulli}(p_{\mathrm{ij}})$.

**Step 6:** Estimate the performance statistics of interest in each validation study by taking the prediction model for the scenario chosen in step 1, $\mathrm{logit}(p_{\mathrm{ij}})=\mu_{\alpha }+\mu_{\beta }\times {\mathrm{age}}_{\mathrm{ij}}$ and evaluating its predictive performance in the generated data from steps 4 and 5.

**Step 7:** Summarise the distribution of the obtained performance statistics across the 1000 studies by calculating summary statistics (such as means and medians) by calculating coefficients of skewness and kurtosis, and plotting histograms.

**Step 8:** Consider transformations of performance statistics if not approximately normally distributed and repeat step 7.

##### Extensions to simulations

Additional simulation settings were also considered. For brevity, we do not include full details but they involved (i) limiting the age range to between −42 and 40 (corresponding to 18 and 100 years if the mean age is 60), (ii) varying the distribution of age across studies, and (iii) including an additional predictor and interaction in the data generating model, to reflect a situation where the model being considered for use in clinical practice has missing predictors.

### 3.2 Simulation study results

#### 3.2.1 C-statistic

**Figure 1.**
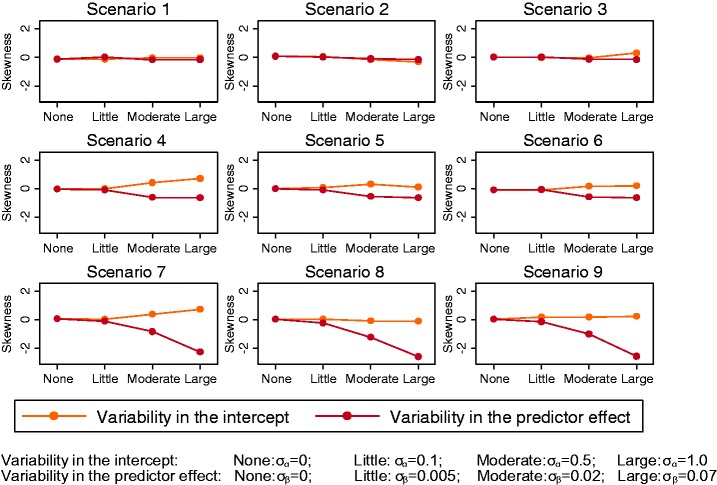
Coefficient of skewness for the *C*-statistic for all scenarios across different simulation settings, with level of variability in either the study-specific intercept *α_j_* or predictor effect *β_j_* along the *x*-axis.

When there was only between-study variability in the intercept term of the data generation model, there was barely any skewness in the observed distribution of the *C-*statistic (Figure 1). Indeed, we found that the width of the distribution was tiny given that there was only minor sampling error (due to the large study sizes) and no between-study heterogeneity in discrimination. However, when the ‘true’ predictor effect in each validation study, β*_j_*, was allowed to vary (settings 5–7), the distribution of the *C-*statistic increasingly deviated from normality as σ_β_ increased. Looking at the coefficient of skewness (Figure 1), the distribution was most skewed in scenarios 7–9, where μ_β_ was larger (stronger predictor on average across studies), which relates to when discrimination was very good (around 0.9 on average) and so the *C-*statistic can only increase slightly to a maximum value of 1 but can decrease much further.

**Figure 2.**
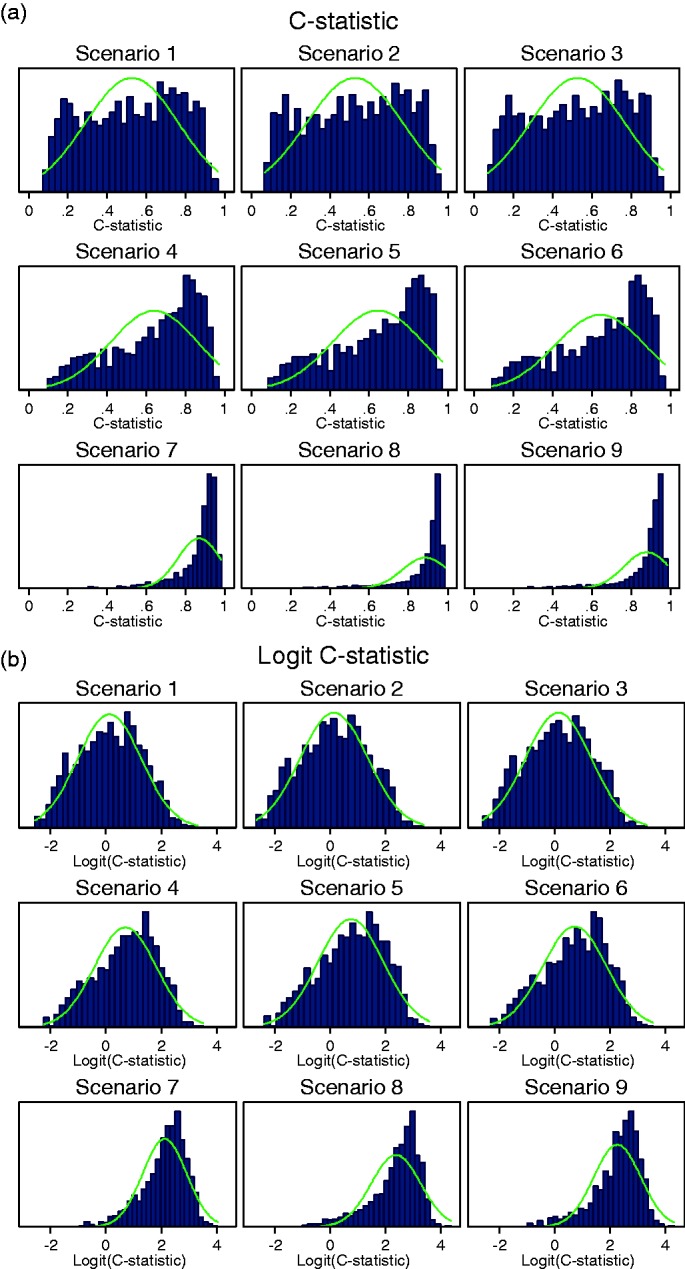
Histograms for (a) the *C*-statistic and (b) logit C-statistic when there is large variability in the predictor effect *β_j_* (setting 7: *σ_β_* = 0.07).

When the variability in β*_j_* was large across studies (setting 7: σ_β_ = 0.07), the *C-*statistic was no longer normally distributed in any of the scenarios (Figure 2(a)). For scenarios 1–3 with a weak predictor, the between-study *C-*statistic was almost uniformly distributed and includes values below 0.5. Such values indicate that the prediction model inversely discriminates between outcome categories in several studies.

##### Transformations of the *C-*statistic

**Figure 3.**
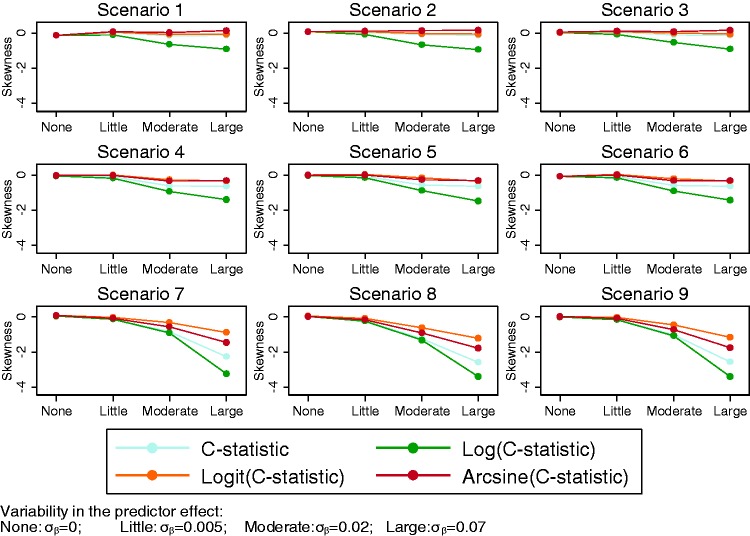
Skewness for the *C*-statistic and transformations of the *C*-statistic for different levels of variability in βj (settings 1, 5, 6 and 7) along the *x*-axis.

Given the apparent non-normality of the *C-*statistic on its original scale in several settings, three transformations (log, logit, arcsine) were considered to ascertain potential improvements in the empirical between-study normality (Figure 3). Results demonstrate that the natural logarithm transformation did not offer any improvement in achieving between-study normality. Some improvements were obtained in terms of skewness when applying the arcsine transformations. However, the logit transformation appeared most advantageous as it greatly reduced the skewness when between-study variability in β*_j_* was large (setting 7: σ_β_ = 0.07, see Figure 2(b)). In addition to this, the between-study distribution of the logit *C-*statistic was no worse in scenarios and settings that were approximately normal on the untransformed scale.

**Figure 4.**
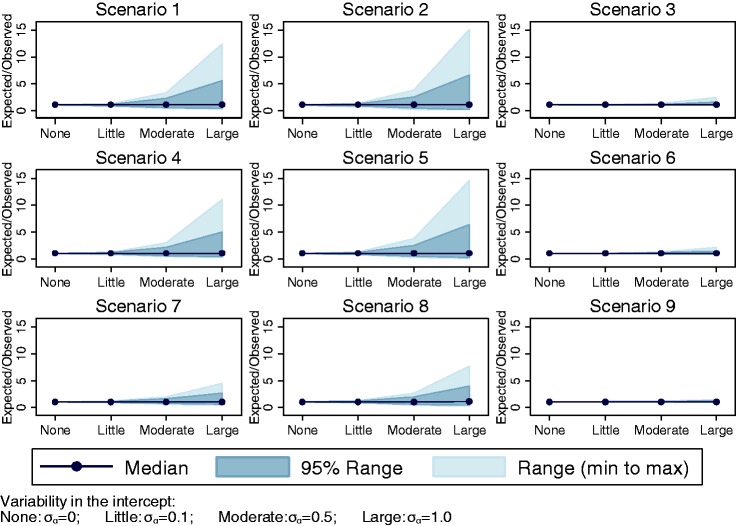
Median and range (95% and minimum to maximum) of values for E/O across different simulation settings with variability in the intercept *α_j_* (settings 1–4).

#### 3.2.2 Ratio of expected and observed proportions

E/O was approximately normally distributed across the validation studies in all scenarios when there was little variability in α*_j_* (setting 2: σ_α_ = 0.1). However, the distributions were more skewed when there was moderate variability in α*_j_* (setting 3: σ_α_ = 0.5) and worse still when there was large variability in α*_j_* (setting 4: σ_α_ = 1.0). The range of E/O also grew larger with increasing heterogeneity in the intercept (Figure 4). The estimated skewness for E/O was 1.32 and 3.00 for scenario 1 in setting 3: σ_α_ = 0.5 and setting 4: σ_α_ = 1.0, respectively.

**Figure 5.**
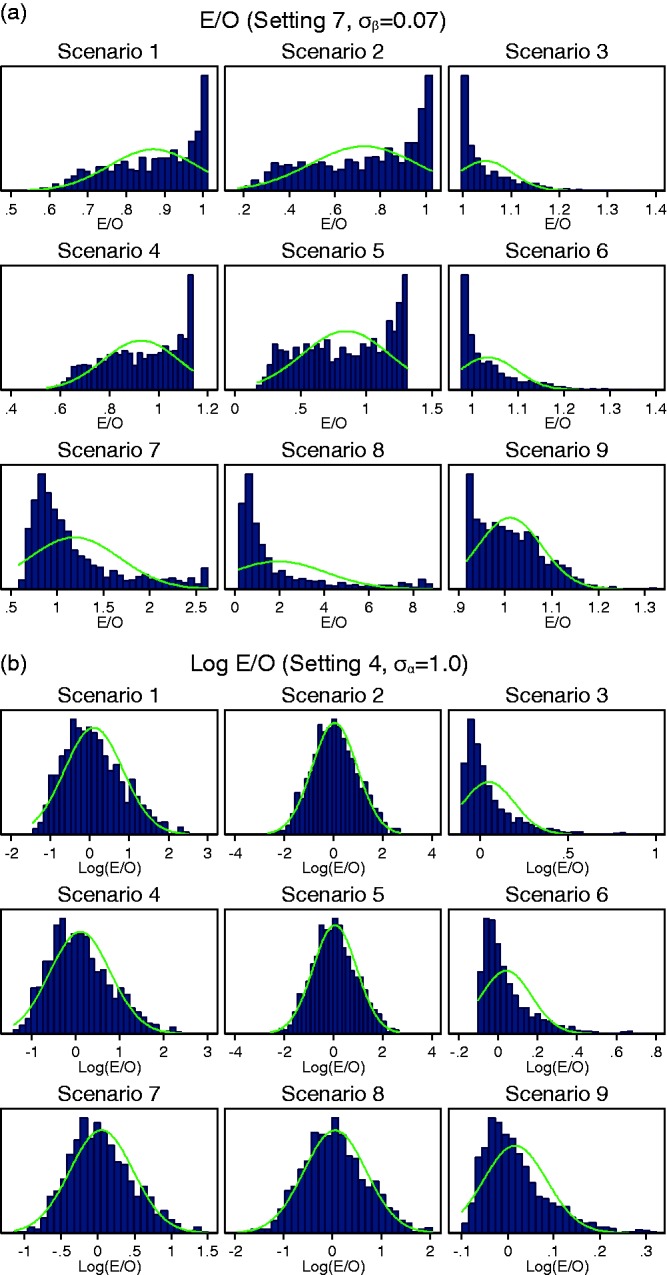
Histograms for (a) E/O in all scenarios when variability in *β_j_* was large (setting 7: *σ_β_* = 0.07), and (b) log(E/O) in all scenarios when variability in *α_j_* was large (setting 4: *σ_α_* = 1.0). Note different *x*-axes used for scenarios.

The distributions of E/O were extremely skewed for most scenarios when variability in *β_j_* was moderate (setting 6: σ_β_ = 0.02) or large (setting 7: σ_β_ = 0.07, Figure 5(a)). The distribution could be skewed in either direction but was often bounded close to one for scenarios 1–3 and bounded at different values for scenarios 4–6. Therefore, the peak of the distribution was often at this boundary value.

##### Transformations of E/O

**Figure 6.**
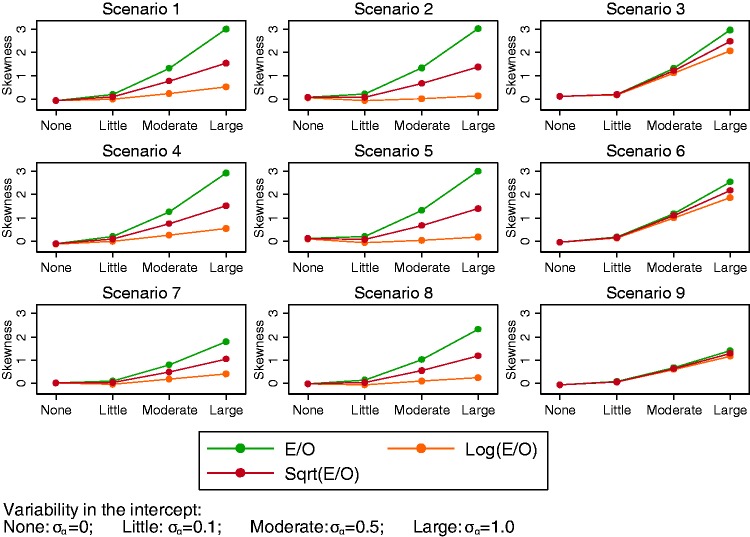
Skewness for E/O and transformations of E/O for different levels of variability in *α_j_* (settings 1–4) along the *x*-axis.

A logarithm transformation applied to E/O improved the shape of distributions when there was variability in α*_j_*, resulting in distributions that were closer to approximate normal distributions (Figure 5(b)). However, the between-study distributions remained skewed for scenarios 3, 6 and 9 where the average intercept was high resulting in a large number of outcomes. The natural logarithm transformation resulted in distributions closer to the normal distribution compared to the square root transformation (Figure 6).

Using the natural logarithm transformation for E/O also improved the shape of the distributions for scenarios 7–9 when there was moderate variability in *β_j_* (setting 6: σ_β_ = 0.02). However, the natural logarithm transformation did not improve the distributions of E/O when variability in *β_j_* was large (setting 7: σ_β_ = 0.07), which remained very skewed in most scenarios (Supplemental material 2). The square root transformation did not improve the distribution of E/O in this setting either.

#### 3.2.3 Calibration slope

The between-study distribution of the calibration slope was approximately normal in all scenarios (1–9) and in all simulation settings for variability in *α_j_* or *β_j_* (settings 2–7). Distributions were fairly symmetrical with the coefficient of skewness ranging from −0.3 and 0.33 across all simulation scenarios and settings (additional figures can be found in Supplemental material 2). Variability in *α_j_* did not affect the distribution of calibration slope, so the distributions were almost identical to setting 1 where there is no variability in *α_j_* or *β_j_*. The SD of the between-study distribution increased as *σ_β_* increased and was larger when *μ_β_* was smaller (see Supplemental material 2).

#### 3.2.4 Calibration-in-the-large

**Figure 7.**
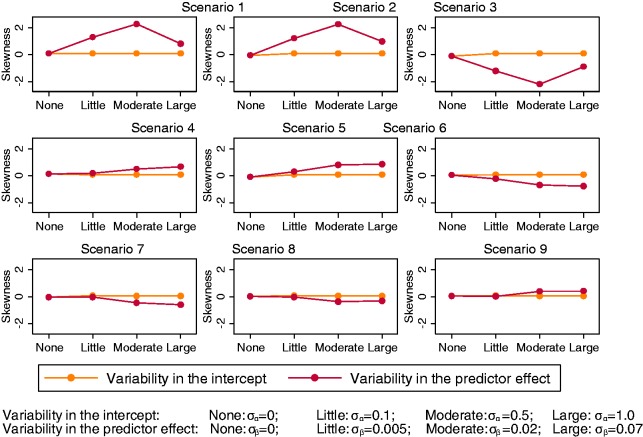
Skewness for the calibration-in-the-large for different levels of variability in *α_j_* or *β_j_* along the *x*-axis.

Calibration-in-the-large was approximately normally distributed and remained symmetrical with coefficients of skewness close to zero in settings 2–4 with variability in the intercept *α_j_*. Variability in the predictor effect *β_j_*, however, did cause the distribution to skew, particularly in scenarios 1–3 where the mean predictor effect was weak (Figure 7).

The width of the distributions for calibration-in-the-large was approximately the same for different scenarios but increased as variability in *α_j_* increased (see Supplemental material 2 for figure). The width of the distribution increases with variability in the predictor effect *β_j_* too, with wider distributions as *σ_β_* increases; however, the distribution also becomes skewed (see Supplemental material 2 for figure). The distributions for all scenarios were positively skewed with the exception of scenarios 3, 6 and 9 (large intercept) which were negatively skewed. The distributions became skewed when the variability was large relative to the value of the average predictor effect *μ_β_*. Therefore, distributions for scenarios 1–3 (weak predictor) were skewed when there was little variability in *β_j_* (setting 5: σ_β_ = 0.005), distributions for scenarios 1–6 were skewed when there was moderate variability in *β_j_* (setting 6: σ_β_ = 0.02) and distributions for all scenarios were skewed when there was large variability in *β_j_* (setting 7: σ_β_ = 0.07). See supplemental material 2 for histograms in setting 6 as an example.

#### 3.2.5 Results of simulation extensions settings

The findings from the previous simulations remained the same in the extensions. In particular, the logit transformation for the *C*-statistic and the natural logarithm transformation for the E/O statistic were helpful to improve normality. Results are available in Supplemental material 3.

## 4 Illustrative example: meta-analysis of the *C-*statistic across practices for QRISK2

The most important finding of the simulation study is that the logit scale is preferable for meta-analysis of the *C*-statistic. We now illustrate the implications of this by examining the discrimination performance of the QRISK2 model across 25 general practices (hereafter referred to as ‘studies’).^25^ The QRISK2 model was developed to predict the 10-year cardiovascular disease risk for use in general practice, in individuals without a prior diagnosis of cardiovascular disease. The model includes the following predictors: ethnicity, age, BMI, Townsend (deprivation) score, systolic blood pressure, cholesterol/HDL ratio, family history of coronary heart disease, smoking status, treated hypertension, type 2 diabetes, rheumatoid arthritis, atrial fibrillation, and renal disease. The model also includes interactions between age and the other predictors. The *C-*statistic was estimated in each study separately, and the standard errors of the *C-*statistic estimate and the standard error of the logit *C*-statistic estimate were obtained using bootstrapping. A random-effects meta-analysis was then fitted using REML to pool the *C-*statistics, either on their original scale or the logit scale. Forest plots are given in Supplemental material 4.

Results (Table 3) demonstrate that there is slightly more heterogeneity (relative to within-study variability) on the original scale compared to the logit scale (*I*^2 ^= 91.2% and *I*^2 ^= 88.6% respectively), and that some studies (e.g. 12 and 24 with *C*-statistics much closer to 1) receive less weight when pooled using the logit scale compared to the original scale. However, the summary *C-*statistic and its 95% confidence interval are very similar when using either the original scale or the logit scale in the meta-analysis (Table 3).

**Table 3.** Random-effects meta-analysis results for the QRISK2 example.

Scale used	Pooled *C-*statistic	95% Confidence interval	95% Prediction interval	*I*^2^ statistic (%)
Original	0.829	0.800–0.859	0.691–0.968	91.2
Logit	0.830	0.800–0.856	0.672–0.921	88.6

To quantify the impact of heterogeneity more meaningfully than *I*^2^, we used equation (6) to calculate an approximate 95% prediction interval for the *C*-statistic in a new setting (back-transforming to the *C-*statistic scale when the logit scale was used). There are notable differences in the prediction intervals depending on which scale we use for pooling. Working on the original *C-*statistic scale leads to a symmetric prediction interval about the summary estimate; however, the use of the logit scale leads to symmetry on the logit scale, but asymmetry on the *C-*statistic scale. Further, the lower and upper values were lower when using the logit scale (95% PI: 0.67 to 0.92) compared to the original scale (95% PI: 0.69 to 0.97). Therefore, although the summary (pooled) estimates of the *C*-statistic are very similar when using the *C*-statistic or logit *C*-statistic scale, the predicted discrimination performance of QRISK2 based on the prediction interval is slightly lower when using the logit transformation.

Partlett and Riley note that the accuracy of equation (6) is most suitable when heterogeneity is relatively large, and there are not a mixture of small and large studies.^33^ To address this, we restricted the studies to those with >20 outcomes, which removed four smaller practices. This is also akin to situations where within-study normality of study estimates is likely to be justified, due to the CLT. The findings were similar to before, with the 95% prediction interval having lower values at the bounds when using the logit-scale (95% PI: 0.67–0.91) compared to the original scale (95% PI: 0.70–0.94).

## 5 Discussion

Summarising how well a certain prediction model performs across different samples, studies, locations or, settings is becoming increasingly of interest in clinical research,^12^ with examples of this being done for prediction models of breast cancer incidence, development of gastric cancer and risk of kidney failure.^37–39^ A random-effects model is appropriate to combine estimates of model performance across multiple validation studies, and to investigate the presence of statistical heterogeneity. Hereby, it is typically assumed that performance measures are normally distributed across studies, which is important when deriving approximate prediction intervals. We investigated this assumption for a variety of scenarios, and the key findings are given in Box 2, and now summarised.

**Box 2. Key findings from the simulation study looking at true between-study distributions of performance statistics.**

***C-*statistic:**

- The between-study distribution of the *C-*statistic was often skewed, especially when there was variability in the predictor effect (*β_j_*) and when the distribution of the predictor values (also known as case-mix) varied across studies.
- The logit-*C* transformation best approximated the Normal distribution, although some degree of skewness remained in the presence of strong predictor effects (scenarios 7–9) or when variability in *β_j_* was large.

**Expected/observed number of outcomes:**

- The E/O had an approximately normal between-study distribution when baseline risk (*α_j_*) was similar across studies. However, it was skewed when baseline risk and/or predictor effects varied across studies.
- The log E/O transformation improved the normality assumption when there was variability in baseline risk and/or predictor effects. Skewness remained when the baseline risk was high (such as in scenarios 3, 6 and 9) or when predictor effects substantially varied across studies.

**Calibration slope:**

- Calibration slope had an approximately normal between-study distribution in all settings considered. Variability in the baseline risk or predictor effect only affected the mean value and SD but not the shape of the distribution.

**Calibration-in-the-large:**

- Calibration-in-the-large had an approximately normal between-study distribution when there was variability in the intercept, although the width of the distribution increased with increasing variability in *α_j_*.
- The distribution was skewed when variability in the predictor effect was large relative to the size of the predictor effect.

An important finding is that the original *C-*statistic scale is inappropriate for use in most random-effects meta-analyses, as the between-study distribution of the *C*-statistic on the original scale is not normally distributed when there is variability in the predictor effect across studies. A simulation study by Austin and Steyerberg showed that the *C-*statistic depends on both the log odds ratio and variance of a continuous explanatory variable.^40^ Our study supports this as when the age distribution was fixed, the *C-*statistic increased as the predictor effect increased (relating to the log odds ratio). The between-study distribution of the *C-*statistic was also wider when the age distribution varied across studies as in the simulation extensions. Variability in the predictor effect across studies is likely to occur in practice when studies include different populations, or adopt (slightly) different definitions for predictors and/or outcomes. However, pooling of untransformed *C*-statistics is still commonplace in many meta-analyses.^15,19^ We found that the logit scale is more appropriate and therefore should be preferred when pooling *C*-statistics. Our QRISK2 example showed that, although the pooled *C-*statistic and confidence interval are relatively unaffected by the scale chosen, the estimated prediction interval can differ importantly when using the logit scale rather than the original scale. This, in turn, may substantially affect any inferences on a model’s potential generalisability.

Despite the advantages of using the logit transformation, the between-study distributions of the *C-*statistic may remain skewed when there is large variability in strong predictor effects. For E/O, the log scale was generally more appropriate but again problems may occur when there is large variability in baseline risk and the incidence of the outcome is high or when there is large variability in predictor effects. The between-study distributions of calibration-in-the-large and the calibration slope were approximately normal in all settings with the exception of when the variability in the predictor effect was large in relation to the size of the predictor effect for calibration-in-the-large. As a ratio, E/O has a lower bound of 0 and no upper bound in theory. Therefore, we expect that E/O will become skewed as between-study heterogeneity in the predictor effect increases, as this will increase the potential for large expected values relative to observed values in some settings. However, in the simulation study we saw the E/O distributions for individual simulation scenarios bounded. This is due to the expected prevalence due to the model combined with the strength of the model predictor and the amount of between-study heterogeneity. Similarly, boundaries were also observed for calibration-in-the-large, but only when there was large heterogeneity in the predictor effect. E/O and calibration-in-the-large are closely related measures, and as such it is preferable to use calibration-in-the-large rather than E/O for meta-analysis. However, when IPD are not available such as when conducting a systematic review and meta-analysis of published validation studies, all relevant performance measures may not be reported in all studies. For this reason, we present recommendations for both E/O and calibration-in-the-large.

The key recommendations for the scale on which to pool performance statistics in a random-effects meta-analysis are given in Box 3.

**Box 3. Recommendations for pooling performance statistics in a meta-analysis to help improve between-study normality.**

The following scales should be used between-studies in a random-effects meta-analysis and subsequently for deriving prediction intervals.

- Use logit transformed *C-*statistics
- Use natural logarithm transformed ratio of expected/observed (E/O) number of outcomes
- Use original scale for calibration slope
- Use original scale for calibration-in-the-large

Caution is warranted when the predictor effects (*β*) and baseline risk substantially vary across studies. The normality assumption between-studies is likely to be less reliable (and therefore calculation of 95% prediction intervals following random-effects meta-analysis may be unreliable under a normal assumption).

Summarising calibration-in-the-large is preferable to E/O. Although the two measures are closely related, the between-study distribution is more likely to be approximately normal for calibration-in-the-large and does not require transformation.

In addition to measures of calibration and discrimination, test accuracy measures such as sensitivity and specificity can be calculated if the model is to be tested as a decision rule (i.e. test positive if patients have a probability greater than some specified value). When meta-analysed, sensitivity and specificity are usually assumed to follow a bivariate normal distribution on the logit scale.^41,42^

Although we considered a large variety of simulation settings, further research is still needed. For example, simulation settings in which the distributions of study-specific intercepts and predictor effects are sampled from a different distribution could be considered. We also mainly considered settings in which the prediction model was correct on average (model parameters were the mean values from which study intercepts and predictor effects were sampled). In reality, the prediction model is itself developed using a smaller sample in which the intercept and/or predictor effect may lie in the tails of the distributions considered rather than the centre. This could also affect the between-study distributions of the performance measures and should be considered in further work. In one of our extensions, we did look at when the true model had an additional predictor that was not included in the developed model, but this did not change our findings. However, further simulations in a wider range of mis-specified true models would be welcome. We only considered a prediction model for a binary outcome, and so further work looking at survival models is also required to examine if our recommendations generalise, and could additionally consider the D-statistic for discrimination.^43^

Further work should also examine the within-study distributions after transforming the performance statistics, and whether the scale for the within-study distributions should be the same or different to those between-studies.^35^ Also, we note that if IPD are not available for all studies or clusters, researchers are limited to the information reported by other authors who may not report standard errors or only report them for the original statistic rather than for any transformed scale. One option is to use the Delta method to obtain the standard error of the transformed statistic^17^; however, we do not know the impact of this on the final prediction intervals and therefore this should also be investigated in further work.

In summary, when performing random-effects meta-analysis of the performance of a particular prediction model that is validated across different study samples or datasets, we found that the logit transformation and the natural logarithm transformation worked best for the *C-*statistic and ratio of expected and observed number of outcomes, respectively, as it improved the normality of between-study distributions substantially, facilitating the derivation of prediction intervals. However, caution should still be taken in situations where large heterogeneity in the predictor effects across studies may be expected, as the normality assumption could still be inappropriate for between-study distributions of performance statistics.

## Supplementary Material

Supplementary material
