# Supplementary material for: Meta-analysis of prediction model performance across multiple studies: Which scale helps ensure between-study normality for the C-statistic and calibration measures?
Source: Stat Methods Med Res. 2017 May 8;27(11):3505–22. doi: 10.1177/0962280217705678 (PMC6193210; doi:10.1177/0962280217705678)
Supplement: Supplementary material [file SMM705678_supplementary_material.pdf]

Supplementary material 1

Preliminary simulations were run to decide how many studies and the sample size for studies that would be sufficient to evaluate the between-study distribution (by minimising the within-study variability).

Supplementary Figure 1 shows histograms for the C-statistic calculated using different sample sizes (rows) and numbers of studies (columns). The size of the samples has a greater impact on the variability of the estimated C-statistic than the number of studies used. Based on the observed graphs, 500000 individuals was selected as an appropriate sample size. The distribution is narrower compared to 100000 individuals, and increasing the sample size to 1000000 individuals only results in a slightly narrower distribution but would increase computation time considerably.

The number of studies was chosen to be 1000. Based on the histograms, 500 would probably be adequate, however the main aim of this chapter is to evaluate the shape of the true distributions for performance statistics, therefore 1000 studies was considered more appropriate to better show the true distributional shape.

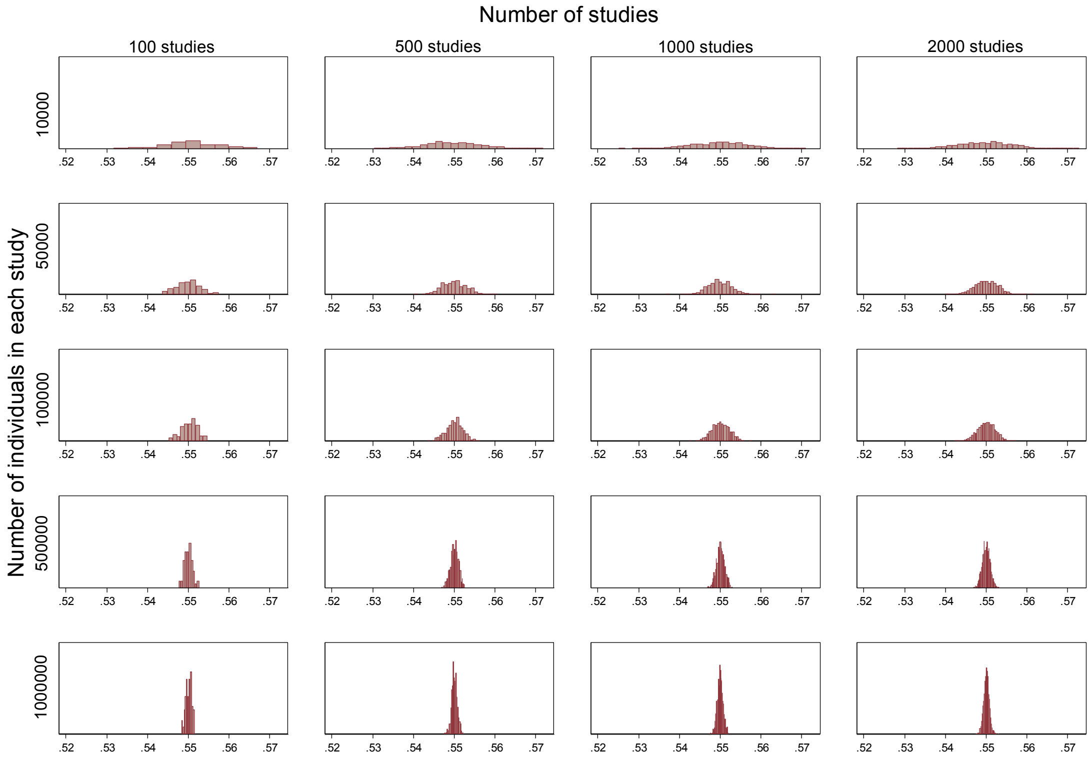

Supplementary Figure 1: Distribution of C-statistic using different number of studies and different number of individuals within each study. Note: columns show different number of studies and rows show different number of individuals within each study.

Supplementary material 2

E/O

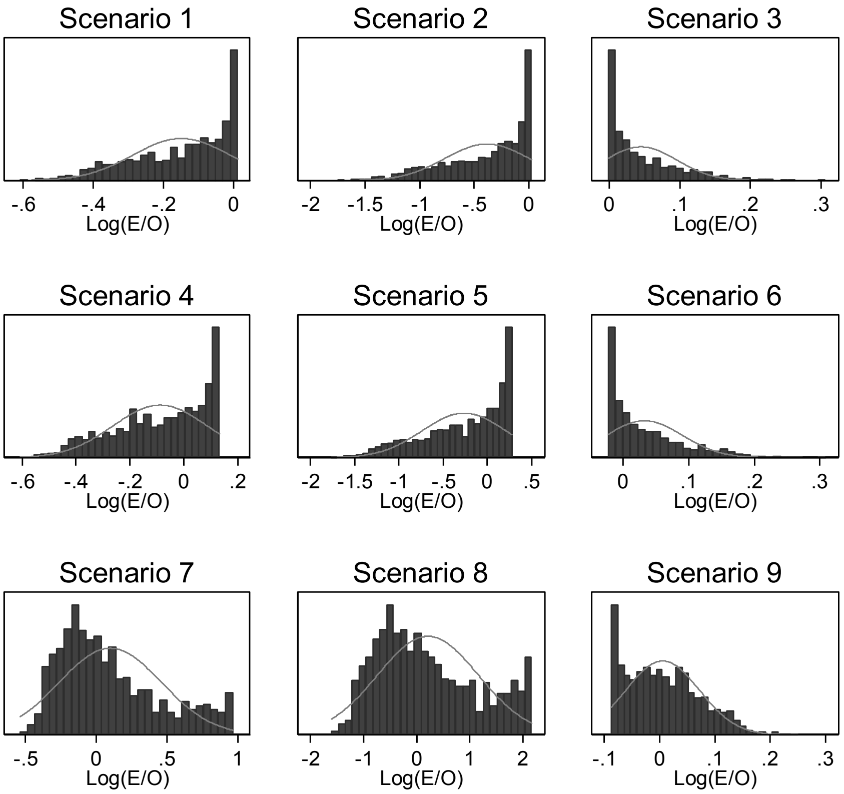

Supplementary Figure 2: Histograms for log(E/O) in all scenarios when variability in β_j was large (setting 7: σ_β=0.07). Note different x axes used.

Calibration slope

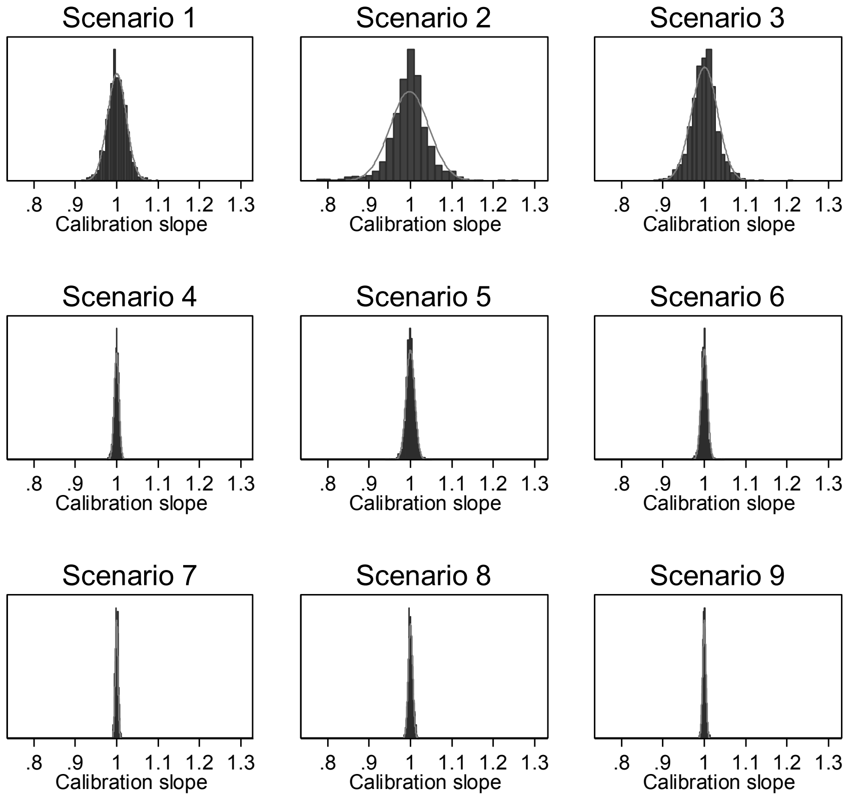

Supplementary Figure 3: Histograms for calibration slope in all scenarios when variability in α_j was large (setting 4: σ_α=1.0).

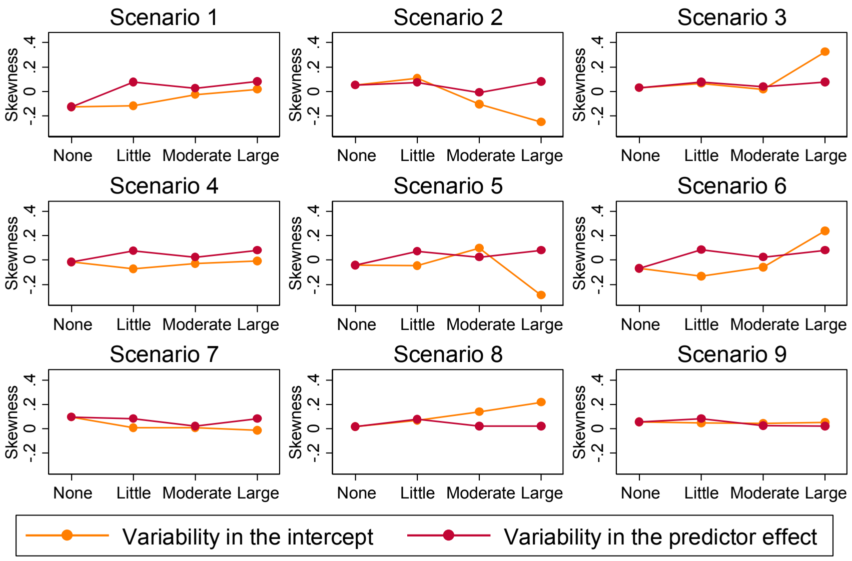

Variability in the intercept: None: σ=0; Little: σ_α=0.1; Moderate: σ_α=0.5; Large: σ_α=1.0
Variability in the predictor effect: None: σ=0; Little: σ_β=0.005; Moderate: σ_β=0.02; Large: σ_β=0.07

Supplementary Figure 4: Skewness for the calibration slope for different levels of variability in α_j and β_j.

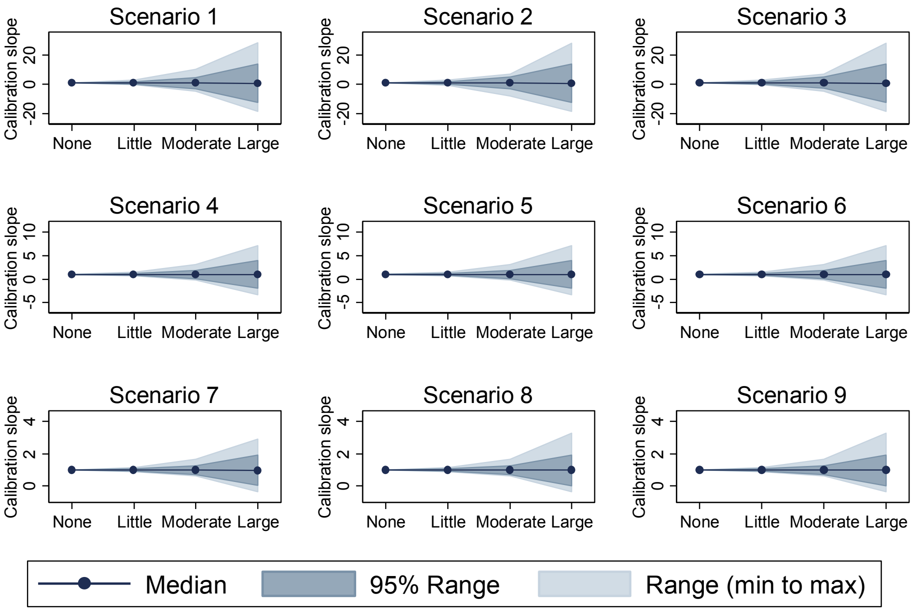

Variability in the predictor effect:
None: σ_β=0; Little: σ_β=0.005; Moderate: σ_β=0.02; Large: σ_β=0.07

Supplementary Figure 5: Median and range of values for calibration slope across different simulation settings with variability in the predictor effect β_j. Note different y axes used.

Calibration-in-the-large

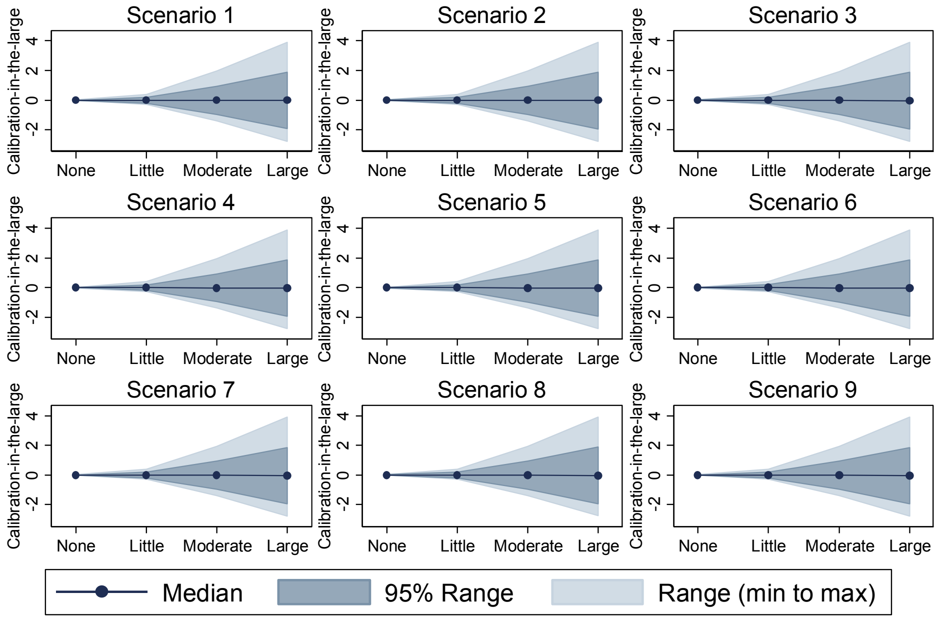

Variability in the intercept:
None: σ_α=0; Little: σ_α=0.1; Moderate: σ_α=0.5; Large: σ_α=1.0

Supplementary Figure 6: Median and range of values for calibration-in-the-large across different simulation settings with variability in the intercept α_j.

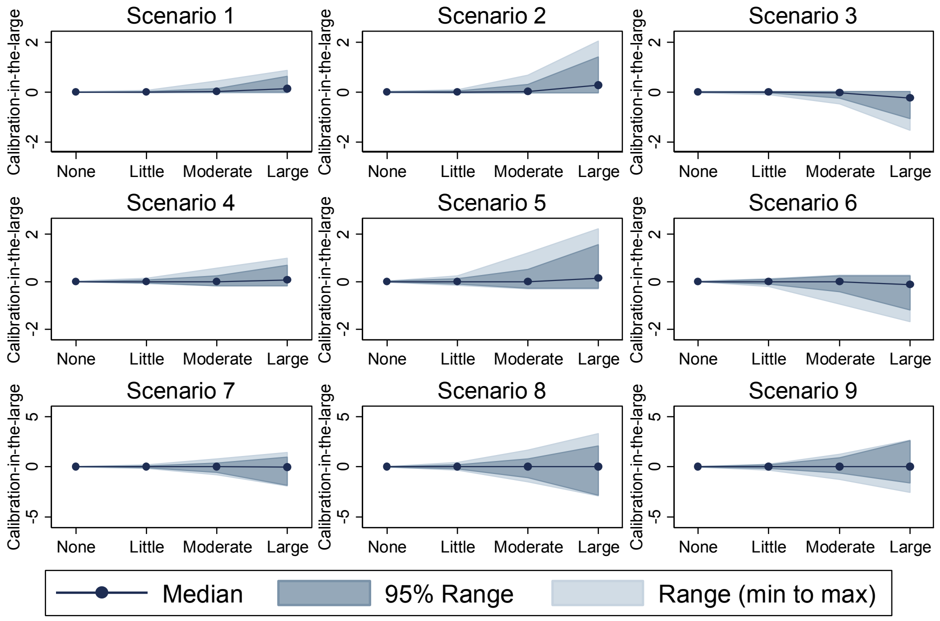

Supplementary Figure 7: Median and range of values for calibration-in-the-large across different simulation settings with variability in the predictor effect β_j. Note different y axes used for scenarios 7 to 9.

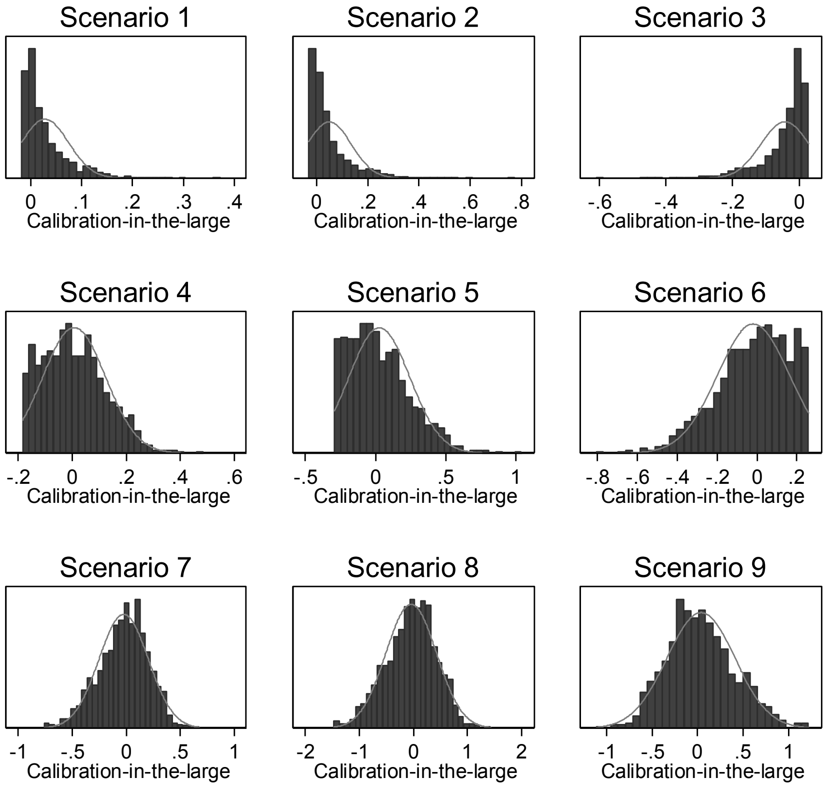

Supplementary Figure 8: Histograms for calibration-in-the-large in all scenarios when variability in β is moderate (setting 6: σ_β=0.02). Note different x axes used.

Supplementary material 3

Simulation extension 1

The values of age sampled for patients were restricted to between -42 and 40 for the mean centred variable (corresponding to between 18 and 100 years if the mean age is 60) to be more realistic. Therefore, if an age<-42 or age>40 was sampled for a patient, age for that patient would be classed as missing and another value would be sampled until an age within the specified range was found.

Deleted: 18

Deleted: years

Deleted: 100

Deleted: years

Restricting the age range did not result in skewed distributions for any of the scenarios. It had very little effect on the distributions at all, except for the C-statistic which was only slightly lower when age was restricted (Supplementary Figure 9).

Deleted: when the predictor was weak (scenario 1-3), but when the predictor was strong (scenario 7-9) the distribution was narrower for E/O and wider for the other three performance measures

Deleted: The average *C*-statistic was also higher than when age was unrestricted.

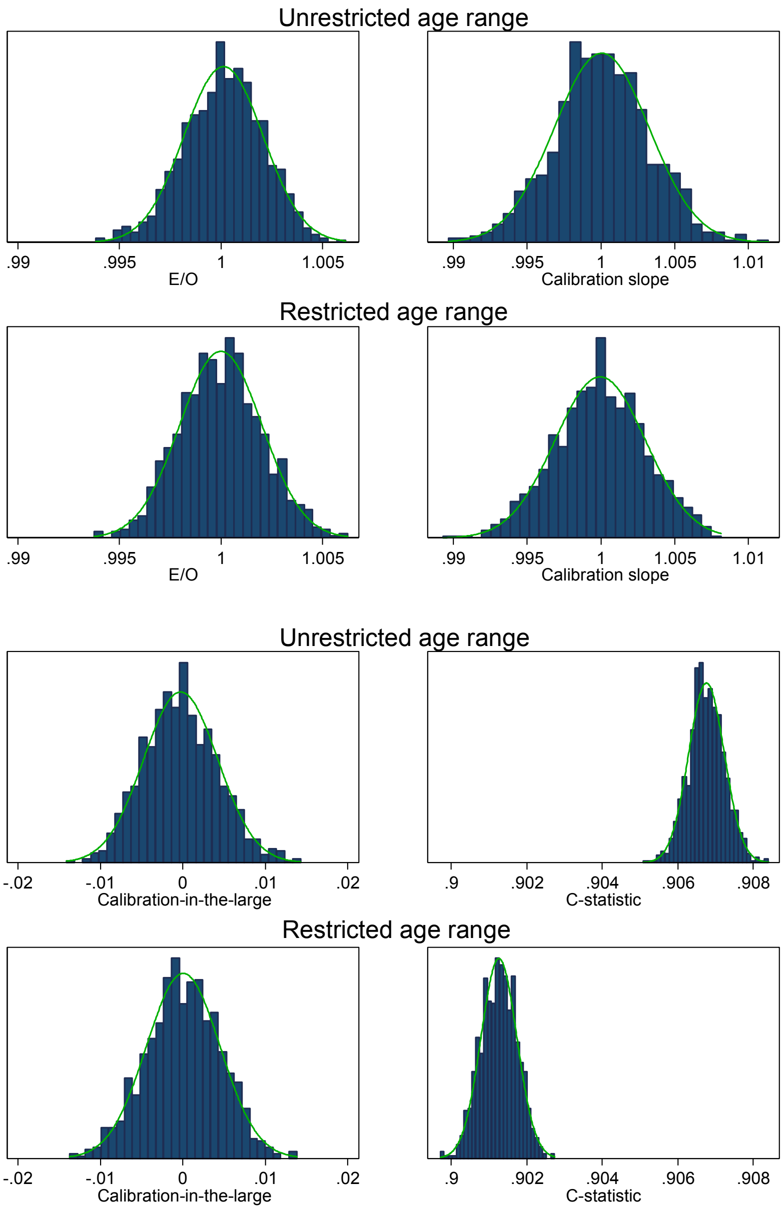

Supplementary Figure 9: Histograms for performance statistics in scenario 7, with data generated using the original mean centred age distribution N(0, 17.62) and age restricted to between -42 and 40(corresponding to 18 and 100 years if the mean age is 60).

Deleted: 6

Deleted: 18

Deleted: 100

Deleted: years

Simulation extension 2

In addition to limiting the age range as in extension 1 above, the distribution from which age was sampled was allowed to vary across studies. Hereto, we sampled the mean and SD values for age using normal distributions. It was assumed that $\text{age}_{ij} \sim \text{Normal}(\mu_j, \sigma_j^2)$, where $\mu_j \sim \text{Normal}(60, 10^2)$ and $\sigma_j^2 \sim \text{Normal}(17.6, 4^2)$.

The between-study distributions of calibration measures (E/O, calibration slope and calibration-in-the-large) were very similar to extension 1 where the range of ages were restricted. However, the between-study distribution for the *C*-statistic was wider when the mean and SD varied and started to skew with strong predictors (such as in scenarios 7 and 8, Supplementary Figure 10). Using the logit transformation offered some improvement towards normality, but distributions sometimes remained skewed. Note that scenario 9 was defined to have a high number of outcomes and a strong predictor, therefore with varying age distributions, computation problems were encountered and performance statistics could not be calculated for all studies. Scenario 9 was excluded as it is likely that for some distributions of age in studies, all patients would have the outcome.

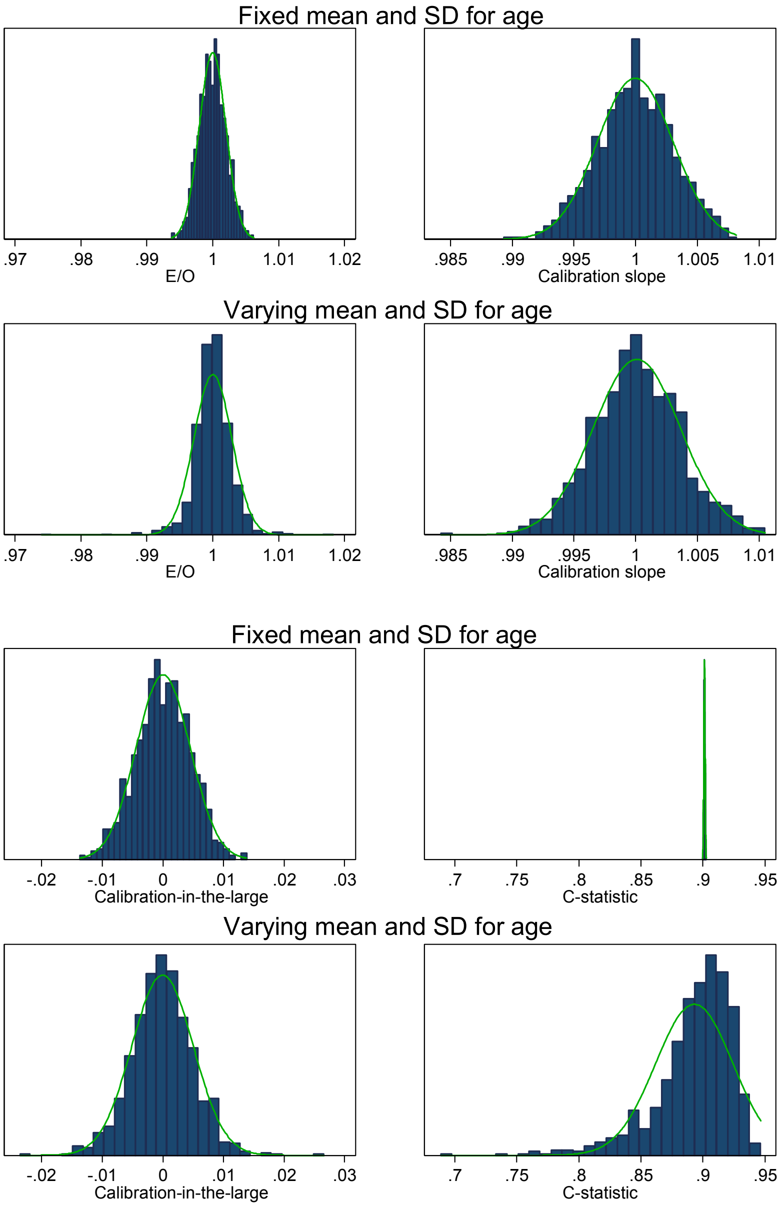

Supplementary Figure 10: Histograms for performance statistics comparing fixed mean and SD for age with random effects on the mean and SD for age in scenario 7.

Simulation extension 3

Additional simulation settings were also considered that involve generating data from a multivariable model that included a second predictor and an interaction between age and the additional predictor. However, the model to be examined for its performance in each study still only included age as in previous simulation settings. Thus, this reflects a situation where the model being considered for use in clinical practice is incomplete (i.e. it misses important predictors), and is therefore a potentially more realistic alternative to those settings described previously. Extension 3 built upon the previous two extensions, so age was restricted and random effects assumed for the mean and SD of age. However for simplicity, no variability in the intercept or predictor effects was considered in this extended setting and simulations were also restricted to scenarios 4 to 6 where the predictor effect was moderate rather than weak (as in scenarios 1 to 3) or strong (as in scenarios 7 to 9). Scenarios 4 to 6 were considered ideal as the original predictor, age, could discriminate reasonably well between patients that have the outcome and patients that do not, but with room for improvement in the model if a further predictor and interaction were added.

Deleted: to between 18 and 100 years

The model for generating data in extension 3 was specified as follows:

$$\text{logit}(p_{ij}) = \alpha + \beta_1 \text{age}_{ij} + \beta_2 \text{pred}_{ij} + \beta_3 (\text{age}_{ij} \times \text{pred}_{ij})$$

This extended setting was considered for both a continuous and a categorical predictor (pred_{ij}). For settings in which pred_{ij} was continuous, the original distribution of age was used for pred_{ij} (not restricting values or allowing the distribution to vary across studies), so values were sampled from $\text{Normal}(60, 17.6^2)$ and the predictor effect assumed to be weak (β_2 =0.01). A correlation of 0.5 was assumed between age and pred so that they were not independent. When the additional predictor was categorical, the predictor prevalence was assumed to be 0.36 (modelling it on sex as a predictor for DVT and using values from Oudega et al.[36] with β_2 =0.1).

Different strengths of interaction effects were considered, depending on whether the additional predictor was continuous or categorical. The values of β_3 were decided by comparing what the probability of the outcome would be with and without the additional predictor and interaction between the two predictors (Supplementary Table 1).

Supplementary Table 1: Defined simulation settings for model with additional predictor and interaction between age and additional predictor.

Simulation setting	OR for additional predictor (OR=exp(β_2))	OR for interaction (OR=exp(β_3))
Extension 3(i)	Continuous, OR=1.01	1.0010
Extension 3(ii)	Continuous, OR=1.01	1.0005
Extension 3(iii)	Continuous, OR=1.01	1.0001
Extension 3(iv)	Categorical, OR=1.1	1.0300
Extension 3(v)	Categorical, OR=1.1	1.0100
Extension 3(vi)	Categorical, OR=1.1	1.0050

The data for the 500 000 patients for each of the 1000 studies was generated, using the new model with specified parameter values, in a similar manner to the steps outlined in Box 1. For the prediction model to be examined, the assumed value of the single coefficient, β_1 would, in reality, also account for some of the variation in the other terms not fitted. Therefore, to calculate its assumed value, a large sample of five million patients was generated to estimate α and β_1 in model (8), and these used to form the prediction model to be examined (Supplementary Table 2). The additional predictor and interaction affected the α values, and the β_1 values were slightly larger only when the missing predictor was continuous.

Deleted: 7

Supplementary Table 2: Parameter values of prediction model to be examined, for extended simulation settings when data are generated including an additional predictor and interaction for scenarios 4 to 6 only (original simulation setting 1 included for comparison).

Scenario	Simulation setting	α	β_1
4	Setting 1: original base scenario	-1.425	0.045
	Extension 3(i): missing continuous predictor & interaction	-1.127	0.050
	Extension 3(ii): missing continuous predictor & interaction	-1.116	0.050
	Extension 3(iii): missing continuous predictor & interaction	-1.125	0.050
	Extension 3(iv): missing categorical predictor & interaction	-1.393	0.045
	Extension 3(v): missing categorical predictor & interaction	-1.390	0.045
	Extension 3(vi): missing categorical predictor & interaction	-1.389	0.045
5	Setting 1: original base scenario	-3.215	0.045
	Extension 3(i): missing continuous predictor & interaction	-2.904	0.050
	Extension 3(ii): missing continuous predictor & interaction	-2.905	0.050
	Extension 3(iii): missing continuous predictor & interaction	-2.904	0.050
	Extension 3(iv): missing categorical predictor & interaction	-3.184	0.045
	Extension 3(v): missing categorical predictor & interaction	-3.176	0.045
	Extension 3(vi): missing categorical predictor & interaction	-3.183	0.045
6	Setting 1: original base scenario	2.440	0.045
	Extension 3(i): missing continuous predictor & interaction	2.719	0.050
	Extension 3(ii): missing continuous predictor & interaction	2.672	0.050
	Extension 3(iii): missing continuous predictor & interaction	2.718	0.050
	Extension 3(iv): missing categorical predictor & interaction	2.486	0.045
	Extension 3(v): missing categorical predictor & interaction	2.488	0.045
	Extension 3(vi): missing categorical predictor & interaction	2.455	0.045

When the missing predictor was categorical, the average performance across studies deteriorated as the strength of the interaction increased for all four performance measures, however the width of the between-study distribution was not affected. When the missing predictor was continuous, the width of the between-study distributions increased slightly for all performance measures apart from E/O where a slight decrease in the width of the distribution was observed. However, the distributions remain relatively normal with little skew (Supplementary Figure 11). The variances of the between-study distributions remained very small and are likely due to the minimal amount of sampling error rather than any between-study heterogeneity.

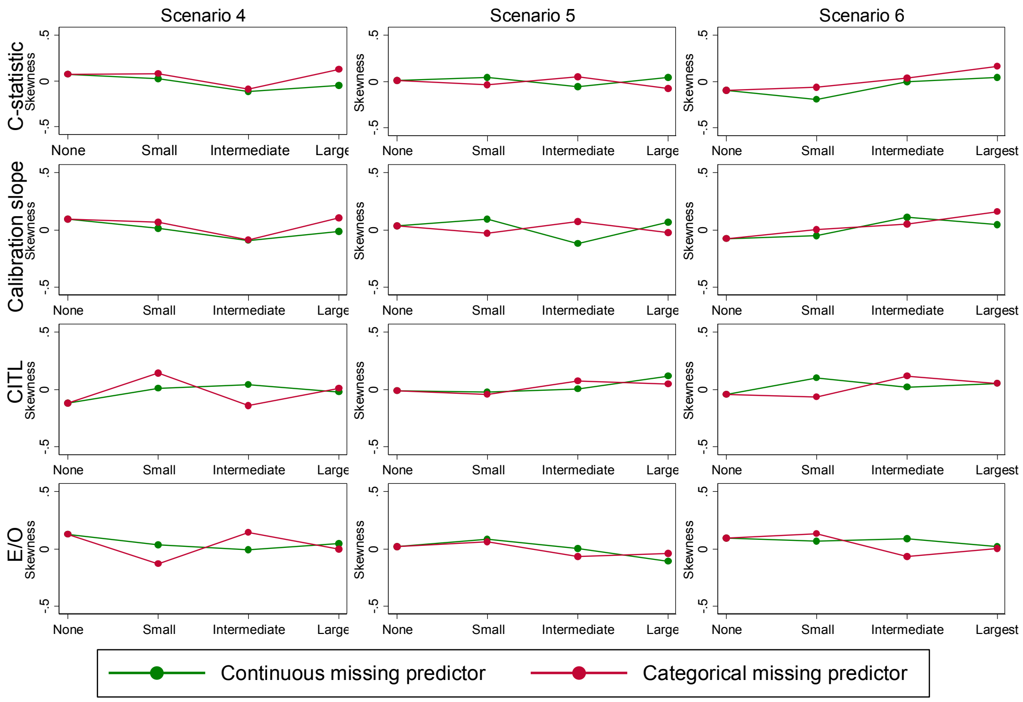

Supplementary Figure 11: Skewness of all performance measures (rows) in scenarios 4 to 6 (columns) for simulation extension 3 where the x axis represents the strength of the interaction effect (small to large) compared to extension 2 where there was no missing predictor or interaction ('none' on x-axis).

Supplementary material 4

Illustrative example

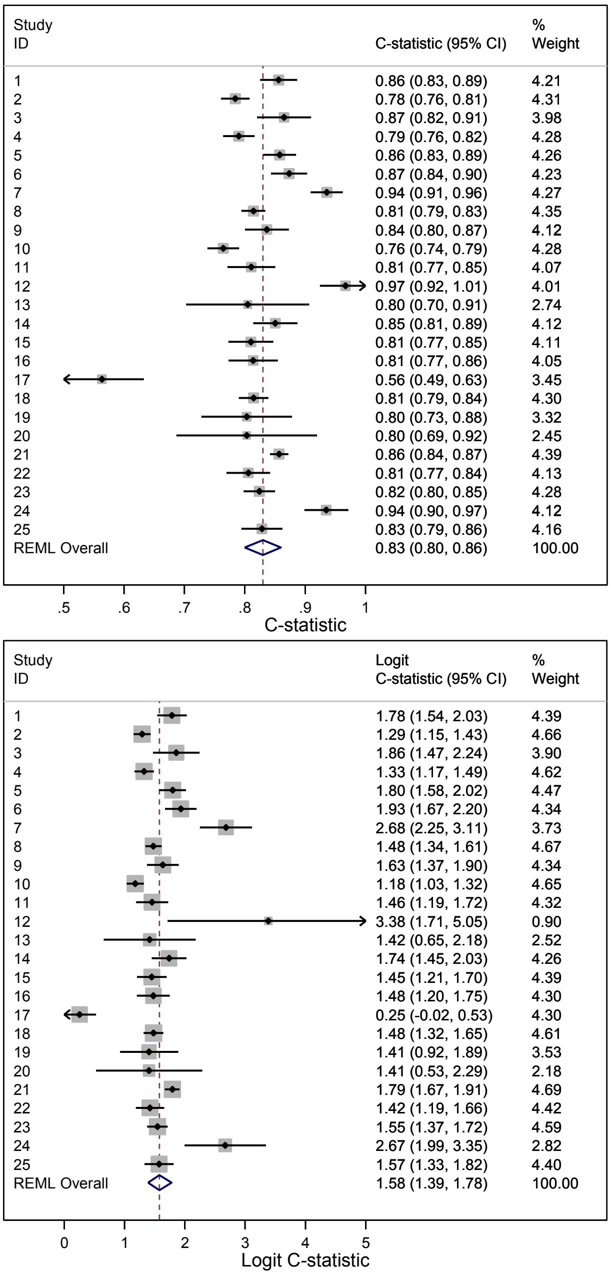

Supplementary Figure 12: Forest plots for meta-analysis of the C-statistic on the original and logit scales.
